# Metastatic Periampullary Tumor from Hepatocellular Carcinoma Presenting as Gastrointestinal Bleeding

**DOI:** 10.1155/2015/732140

**Published:** 2015-04-29

**Authors:** Amir Kashani, Nicholas N. Nissen, Maha Guindi, Laith H. Jamil

**Affiliations:** ^1^Department of Gastroenterology, Cedars-Sinai Medical Center, 8700 Beverly Boulevard, Los Angeles, CA 90048, USA; ^2^Hepatobiliary and Pancreatic Surgery, Cedars-Sinai Medical Center, 8700 Beverly Boulevard, Los Angeles, CA 90048, USA; ^3^Department of Pathology and Laboratory Medicine, Cedars-Sinai Medical Center, 8700 Beverly Boulevard, Los Angeles, CA 90048, USA

## Abstract

Periampullary tumors constitute a number of diverse neoplastic lesions located within 2 cm of the major duodenal papilla; among these, metastatic lesions account for only a small proportion of the periampullary tumors. To our knowledge, a metastatic periampullary tumor from hepatocellular carcinoma has never been reported. A 62-year-old male reported to our institute for fatigue and low hemoglobin. His medical history was remarkable for multifocal hepatocellular carcinoma (HCC) treated with selective transcatheter arterial chemoembolization (TACE). An esophagogastroduodenoscopy (EGD) was performed which revealed a periampullary mass. Histopathology was consistent with metastatic moderately differentiated HCC. Two endoloops were deployed around the base of the mass one month apart. The mass eventually sloughed off and patient's hemoglobin level stabilized. We postulated that periampullary metastasis in this patient was the result of tumor fragments migration through the biliary tracts and that TACE which increases tumor fragments burden might have played a contributory role. Metastasis of HCC to the gastrointestinal (GI) tract should be considered as a cause of GI bleeding.

## 1. Introduction

Common sites of metastases from hepatocellular carcinoma (HCC) are the lung, bone, lymph nodes, and adrenal glands [1]. Gastrointestinal (GI) metastases from HCC are rare, with most cases reported in the stomach and duodenum [2]. The suggested mechanism of metastasis is mainly direct invasion of a tumor contiguous with the GI tract [2]. In this paper, we describe a patient with an isolated metastatic periampullary tumor from HCC who presented with GI bleeding. This finding is unusual for two reasons: first, HCC does not tend to metastasize to the small bowel and, second, metastatic periampullary tumors are very uncommon [2, 3]. We also will elaborate on the possible pathogenesis of periampullary metastasis in our patient.

## 2. Case Report

**Figure 1:**
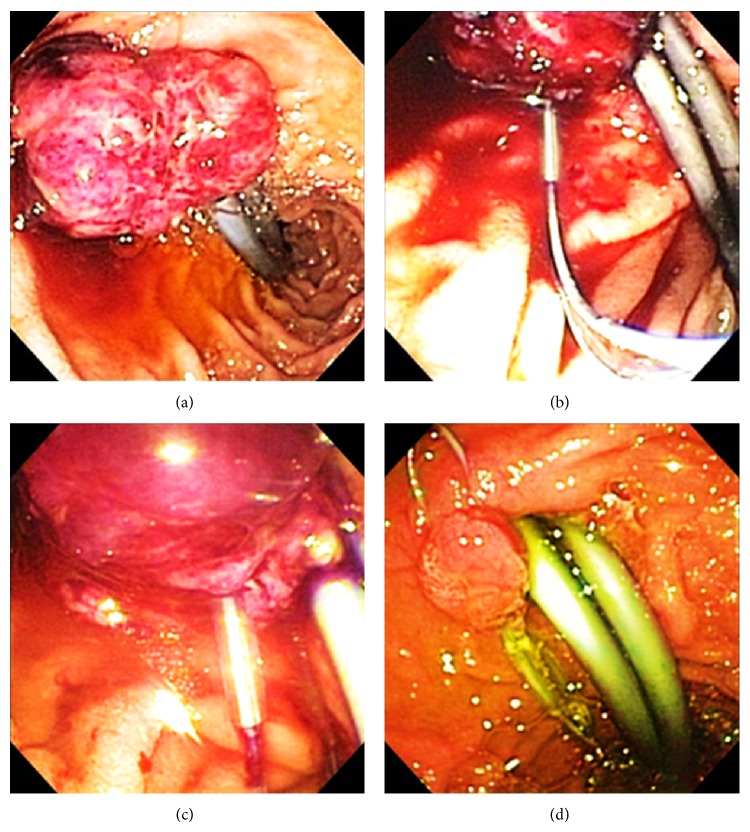
(a) A 3 × 2 cm ulcerated periampullary mass; previous biliary stents in place. ((b), (c)) First and second endoloops were deployed at the base of the mass one month apart. (d) Tumor remnant tissue one month after the second endoloop; two endoloops are still in place.

**Figure 2:**
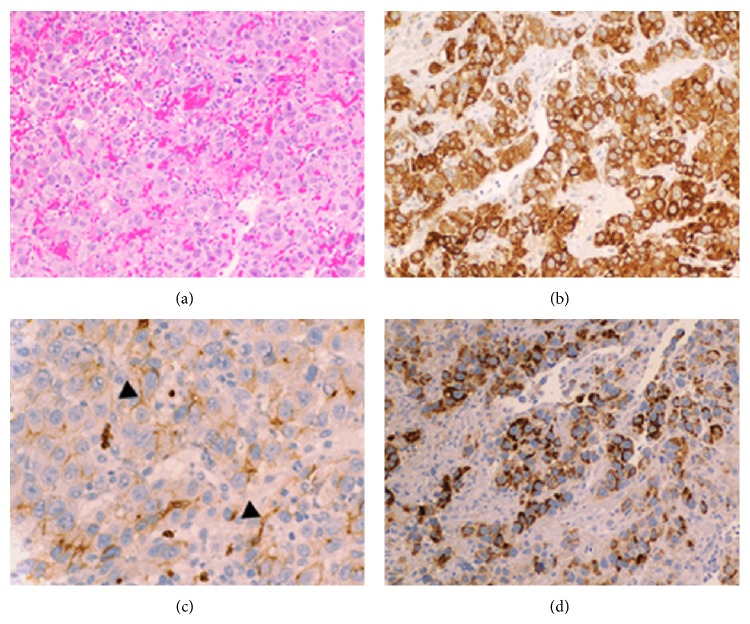
(a) (H&E, 20×) Poorly differentiated hepatocellular carcinoma composed of large epithelial cells with abundant pink cytoplasm and central atypical nuclei. (b) (H&E, 20×) Glypican-3 immunostain positive (brown chromogen). (c) (H&E, 40×) Polyclonal carcinoembryonic antigen immunostain highlights a positive canalicular pattern (arrowheads). (d) (H&E, 40×) Hepatocyte paraffin-1 immunostain (brown chromogen).

**Figure 3:**
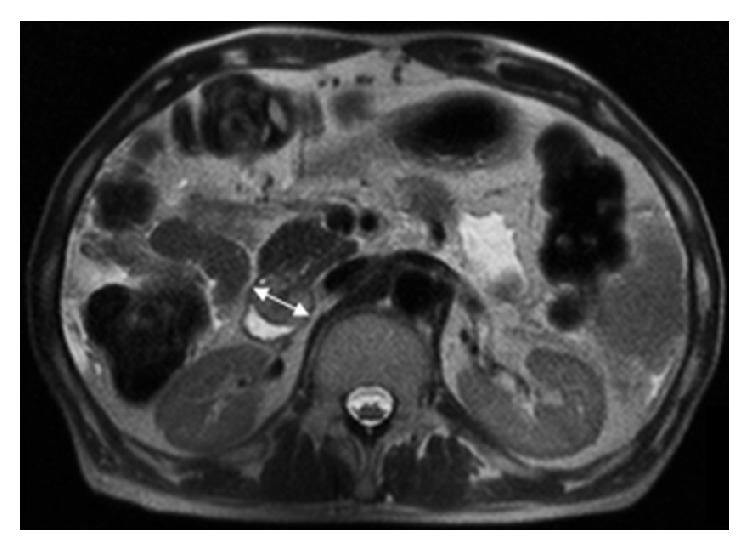
Abdominal magnetic resonance imaging shows a pedunculated mass at the level of ampulla with mild enhancement (double arrow).

A 62-year-old male presented to our institute with fatigue. His medical history was notable for cirrhosis secondary to chronic hepatitis C, complicated by multifocal hepatocellular carcinoma (HCC), which had been lately diagnosed following an episode of obstructive jaundice. Later he underwent multiple biliary interventions including sphincterotomy, dilatation, and stent placement due to recurrent strictures. Cytopathology of the biliary materials had revealed necrotic tissue containing HCC cells. Also, a few months prior to the current presentation, he underwent selective transcatheter arterial chemoembolization (TACE) of three discrete areas of the tumor within the right hepatic lobe. His blood laboratory test upon presentation showed a hemoglobin level of 5.1 g/dL (reference value: 11.6–15.4 g/dL), so he was admitted for further evaluation. An esophagogastroduodenoscopy (EGD) was performed, which revealed columns of grade II and III esophageal varices (EVs), in addition to a 3 by 2 cm periampullary mass at the site of prior sphincterotomy with no sign of active bleeding (Figure 1(a)). This mass was not evident on any of the several previous endoscopic procedures. Histopathology confirmed this mass as a metastatic lesion showing the characteristics of moderately differentiated HCC (Figure 2(a)). Immunohistochemistry studies were positive for hepatocyte paraffin-1, glypican-3, and carcinoembryonic antigen (polyclonal) (Figures 2(b), 2(c), and 2(d)). Thereafter, an extensive metastatic workup was performed which did not reveal any metastatic sites other than the periampullary mass seen on abdominal magnetic resonance imaging (Figure 3). Due to ongoing blood loss, a series of empiric endoscopic band ligations of the EVs were performed until the varices were resolved; however, the patient's hemoglobin level continued to decline. On repeat EGD, the periampullary mass was visualized with adherent blood clots. An endoloop was then deployed around the base of the mass (Figure 1(b)). However, the patient's hemoglobin gradually continued to drop. One month later, a second EGD showed evidence of only minimal necrosis; thus a second endoloop was deployed (Figure 1(c)). One month later, a follow-up endoscopy showed the mass had sloughed off with only slight remnant tissue (Figure 1(d)). Subsequently, the patient's hemoglobin level stabilized without any further blood transfusion required. However, he expired a few months later due to liver failure.

## 3. Discussion

Periampullary tumors constitute a number of diverse neoplastic lesions located within two cm of the major duodenal papilla [4]. Among these, metastatic lesions constitute only a small proportion of periampullary tumors [3]; melanoma, choriocarcinoma, liposarcoma, squamous carcinoma of the lung, and renal cell carcinoma are the reported origins of these metastatic tumors [3, 5]. Although duodenal metastasis from HCC is occasionally reported, to our knowledge an HCC-originating metastatic tumor, involving the periampullary region, has never been reported in the literature. In a postmortem study in patients with HCC, duodenal metastasis is reported in 4% of cases [6]. Meanwhile, premortem metastasis to the duodenum is limited only to a few case reports and series [7–9]. The main suggested route of metastasis is direct invasion of a bulky HCC tumor adjacent to the GI tract [2]. Hence, duodenal invasion is generally seen when the tumor involves the right hepatic lobe, which is in proximity to the first portion of the duodenum [7]. Anatomically, left lobe tumors tend to invade the stomach [10]. The direct invasion can be confirmed by abdominal imaging modalities such as magnetic resonance imaging or computed tomography scan [7, 11]. A less common mechanism of metastasis is hematogenous/lymphatic dissemination [8, 9, 12]. This route of metastasis is tentatively suggested when a direct invasion is excluded and remote metastases to the other organs have been revealed by imaging studies [8]. The presence of portal vein thrombosis is known as a strong indicator of presumed hematogenous spreading of HCC [2]. Due to anatomic correspondence, in which a contiguous liver tumor is invading the duodenum, the proximal duodenum is generally involved, while in hematogenous metastasis involvement of the distal duodenum is also reported [8]. In the absence of remote metastases, isolated duodenal involvement via a hematogenous route is extremely rare [2, 9]. Herein we report a novel case of isolated periampullary metastasis from HCC. In the absence of radiologic evidence of HCC direct invasion to the duodenum, the possibility of this route of metastasis is excluded. Also, imaging modalities did not reveal any evidence of remote metastases, lymphatic involvement, or portal vein thrombosis. Therefore, the possibility of hematogenous/lymphatic dissemination was not deemed high. Involvement of the biliary tracts by cancerous cells is an uncommon finding, which is reported in only 4% of patients with HCC [6]. This can lead to biliary obstruction via impacting the major duodenal papilla with tumor fragments [13]. Moreover, migration of the tumor fragments through the biliary system, although rare, is a suggested route of metastasis in primary liver malignancies [14]. In the present patient, cytology result of the biliary material aspiration on multiple interventions that had been performed due to obstruction confirmed the presence of malignant HCC cells. We postulated that metastasis in our patient was due to invasion of HCC into the biliary ducts, leading to recurrent strictures, which required multiple biliary interventions; this subsequently caused cancerous cells to spread to the periampullary region. Moreover, performing TACE is known to cause tumor necrosis, which leads to an increase in the burden of tumor fragments in the biliary system [15]. In the current patient, it is presumed that TACE might stand as another contributing factor to periampullary metastasis.

Gastrointestinal hemorrhage in patients with HCC is predominantly originating from EVs; gastrointestinal metastases account for about 5% of GI bleeding in these patients [16]. Duodenal metastatic lesions from HCC, although rare, usually manifest as upper GI bleeding, which might be obscured in the context of concurrent cirrhosis and variceal bleeding [7]. In our patient, several band ligations of EVs were performed; however these did not stop the drop in the patient's hemoglobin level. Only after endoscopic treatment of the periampullary tumor was the patient's hemoglobin stabilized. So in patients with HCC and GI bleeding where metastatic lesions to the GI tract are found, these should be considered as the possible source of hemorrhage, especially when bleeding EVs or other sources are not evident. In addition to supportive care, surgical or endoscopic interventions and transarterial embolization are the suggested therapeutic options for bleeding GI metastatic lesions from HCC [7]. Due to the unique location of the tumor in the current patient, we were able to control the bleeding with endoloops which cut off tumor blood perfusion leading to tumor necrosis.

In conclusion, we described a novel case of periampullary metastasis from HCC which presented as GI bleeding. The biliary system is a possible route of metastasis to the periampullary region in patients with liver malignancies. In HCC patients presenting with GI bleeding, metastasis to the GI tract from HCC should be considered as a possible source.
